# Identifying network state-based Parkinson’s disease subtypes using clustering and support vector machine models

**DOI:** 10.3389/fpsyt.2025.1453852

**Published:** 2025-02-13

**Authors:** Benedictor Alexander Nguchu, Yifei Han, Yanming Wang, Peter Shaw

**Affiliations:** ^1^ Oujiang Laboratory (Zhejiang Lab for Regenerative Medicine, Vision and Brain Health), Wenzhou Medical University, Wenzhou, Zhejiang, China; ^2^ School of Biomedical Engineering, School of Ophthalmology and Optometry and Eye Hospital, Wenzhou Medical University, Wenzhou, Zhejiang, China; ^3^ Center for Biomedical Imaging, University of Science and Technology of China, Hefei, Anhui, China

**Keywords:** Parkinson’s disease, PD heterogeneity, PD subtypes, APOE genotype, clustering algorithm, machine learning models

## Abstract

**Introduction:**

Parkinson’s disease (*PD*) heterogeneity poses challenges to the current development of discovering the best therapeutic targets.

**Methods:**

Here, we employ K-means and hierarchical clustering algorithms on data from the Parkinson’s Progression Markers Initiative (*PPMI*) to identify network-specific patterns that describe PD subtypes using the optimal number of brain features. The features were specifically the gray matter volume and dopaminergic features of the neostriatum, i.e., the caudate, putamen, and anterior putamen. We use machine learning (*ML*) algorithms, including Random Forest, Logistic Regression, and Support Vector Machine, to evaluate the diagnostic power of the brain features and network patterns in differentiating the *PD* subtypes and distinguishing *PD* from *HC*. Finally, we assessed whether *PD* subtypes described through network-specific patterns are dependent on the *APOE* genotype.

**Results:**

Using data from 2396 subjects, we show that *PD (n=2037)* is highly associated with APOE ϵ2/ϵ4. Our findings reveal a significant DAT deficit in the left and right structures of the caudate, putamen, and anterior putamen in subjects with *PD* compared to subjects with *SWEDD(n=137) or HC(n=222)*, and that APOE ϵ2/ϵ4 may accelerate DAT deficits and brain alterations in both PD and SWEDD. Furthermore, clinical symptoms of PD in subjects (SWEDD), which hardly validated by DAT scan data, can be explained by variations in APOE genotypes and other brain features beyond DAT. We show the existence of three networks states for the whole data, with the first network state describing the subjects in HC, while the remaining two network states describing the two PD subtypes—one network state typified by a mildly sparsely connected network (patterns) and the other network state characterized by a more intensified sparsity in their network. We also show that the two subtypes of PD are characterized by distinctly different levels of total gray matter volume and DAT deficit. ML models show that features extracted from brain structure and network patterns can serve as reliable biomarkers for PD and its subtypes, with the highest performance (100% AUC, 99.3% accuracy, 0.993 F1) demonstrated by the fine-tuned SVM model.

**Conclusion:**

Our findings suggest that, while PD is generally associated with a larger DAT deficit in specific brain structures of the neostriatum, it exhibits intrinsic heterogeneity across individuals, which may stem from genetic factors. Such heterogeneity can be characterized by ML models and optimally mapped into network states, providing new insights to consider when developing personalized drugs.

## 1 Introduction

Parkinson’s Disease (*PD*) affects about 1% of people over 60 years old and 4 to 5% of those over 85 years old (1), with an incidence of 10 to 20 new cases per 100,000 annually (2). Economically, *PD* incurs billions in direct medical costs and significant indirect costs from lost productivity and caregiving (2). By 2030, *PD* prevalence could double to 14 million, with healthcare costs potentially exceeding tens of billions globally, stressing the need for effective treatments (2, 3). The economic impact includes substantial indirect costs, affecting *GDP* and societal well-being, highlighting the importance of innovative healthcare interventions (3).

Efforts have been made to understand the mechanisms of Parkinson’s disease (*PD*) and the possibilities of identifying the best therapeutic target. However, these efforts face challenges for several reasons. First, *PD*, as a neurodegenerative disorder, has a wide range of clinical manifestations and purported underlying causes, making it difficult to identify common mechanisms and develop therapies that are effective for all patients. Second, studies have demonstrated that PD is not caused by a single factor but rather a combination of several factors, including genetic, environmental, aging, and lifestyle factors (4). This makes it challenging to pinpoint the specific pathways involved in disease evolution. Third, *PD* worsens over time, with different trajectories—some patients exhibit fast progression, while others display slow progression—adding complexity to the overall understanding of the disease and target areas for developing drugs. Therefore, this study aims to provide insights into optimal states (*PD* subtypes) at which patients with *PD* exist, which would guide individuals developing drugs.

Here, we employ three approaches: First, we use clustering approaches—both K-means and hierarchical clustering algorithms—to identify the key states at which *PD* manifests using the optimal number of brain features. Second, we build structural networks of these states (*PD* subtypes) to observe how the brain structures appear in the network space and use this information to describe the *PD* subtypes. Third, we use Machine Learning (*ML*) algorithms to identify the diagnostic powers of brain structures and other features derived from this structural information in differentiating *PD* from *HC* and in differentiating patterns associated with different *PD* subtypes. In addition, we conducted an intensive exploration to identify which *APOE* genotypes are highly associated with *PD* and its subtypes, considering that a significant body of research has shown an association between *PD* and *APOE* genotypes (5) but not at the *PD* subtype scale.

Presently, significant progress in understanding *PD* mechanisms has been made through genetic studies, revealing key mutations such as *SNCA* and *LRRK2*, and large-scale genome-wide association studies identifying multiple risk loci (6). Pathophysiological insights have highlighted the roles of alpha-synuclein aggregation, mitochondrial dysfunction, oxidative stress, and neuroinflammation in *PD* progression (7–9). Some ideas point out that cellular pathways involving autophagy-lysosome dysfunction and endoplasmic reticulum stress may be involved in PD development (10). Neuroanatomical studies emphasize nigrostriatal degeneration and non-motor symptoms, such as cognitive decline, as central to *PD* pathology (11). However, despite these advances, it is still not clear what triggers the alpha-synuclein misfolding, or what are the detailed mechanisms linking mitochondrial dysfunction to selective neuronal vulnerability, and/or what factors accelerate the variability in disease progression among patients. Nevertheless, we hypothesize that Machine learning techniques may offer insights at least to the dimensions of *PD* manifestations and provide intuitions of possible optimal *PD* subtypes, which could aid in targeted drug discovery and treatment based on individual disease profile.

## 2 Method

### 2.1 Data source

The datasets utilized for this study were retrieved from the Image and Data Archive (IDA) database under Parkinson’s Progression Markers Initiative (PPMI, https://ida.loni.usc.edu/login.jsp?project=PPMI, retrieved on Sept.3.2023). They include the data collected directly from the clinics, remote platforms, and online sources. Subjects had a complete set of clinical trials, MRI scanning, demographics, and genetic markers recording. Part of the subjects were Healthy Control (HC), while others had Scans Without Dopaminergic Deficit (SWEDD), and others had moderate Parkinson’s Disease (PD).

### 2.2 Demographics

All participants in the PPMI study belong to four cohorts: PD, HC, SWEDD, and Prodromal Cohort. The number of patients in each cohort was counted and segmented accordingly. A subset of the patient’s personal, genetic, treatment information, and brain data was extracted and merged according to patient number and return visit time. Since some patients have missing data, rows with missing and duplicate values need to be removed during the merging process. Finally, data from all cohorts were combined into a comprehensive dataset. PD subjects were 30 years of age and above, untreated with PD medications (levodopa, dopamine agonists, MAO-B inhibitors, or amantadine), and within 2 years of diagnosis. *Criteria from Clinical features:* They must have Hoehn and Yahr < 3 (only affected in walking or fine motor skills), resting tremor (shaking while at rest), bradykinesia (slowness of movements and difficulty in initiating movements), rigidity (increased resistance, regardless of speed, to passive movements throughout the range of the joint), or a single asymmetric resting tremor or asymmetric bradykinesia. In addition to the clinical features of the disease, PD subjects who had dopamine transporter (DAT) or vesicular monoamine transporter 2 (VMAT-2) demonstrating dopaminergic deficit (uptake/binding reduction, reflecting loss or dysfunction of dopaminergic neurons) consistent with PD, were enrolled as PD cohort. And the subjects who were clinically considered as potential PD but had DAT or VMAT-2 scans without evidence of dopaminergic deficit (SWEDD) were enrolled as SWEDD cohort. Subjects considered HC were those aged 30 years and older, without an active, clinically significant neurological disorder or a first-degree relative with PD.

### 2.3 MRI imaging

MRI imaging for data was performed using a standard protocol electronically shared across all ten sites. The MRI machines used were either Trio™ or Verio™ systems from Siemens Healthcare Systems. The imaging protocol includes sequences for anatomical and diffusion data. Imaging anatomical details was performed through T1-weighted image via a 3D magnetization prepared rapid gradient echo (MPRAGE) sequence, with the following configurations: TR/TR/TI = 2300/3/900ms; 1 mm isotropic resolution; twofold acceleration; sagittal-oblique angulation. Imaging for diffusion was performed using a cardiac-gated 2D single-shot echo-planar DTI sequence, with the following configurations: TE = 88ms, 2 mm isotropic resolution; 72 contiguous slices each 2mm thick, twofold acceleration, axial-oblique aligned along the anterior-posterior commissure; diffusion-weighting gradients along 64 sensitization directions; a b-value of 1000s/mm2; TR in order of 8,400-8,800ms, depending on the subject’s heart rate. After data acquisition, each site transferred the data to the PPMI Imaging Core Lab for checking parameter consistency across data and for processing.

### 2.4 Dopamine transporter and vesicular monoamine transporter 2 imaging and quantification

All PD subjects underwent dopamine transporter (DAT) imaging or vesicular monoamine transporter 2 (VMAT-2) imaging to identify if the clinical features of PD align with dopaminergic features of the brain. The imaging for DAT (i.e., presynaptic protein highly concentrated in the striatum dopaminergic neurons) was performed with the assistance of 123I Ioflupane (also called FP-CIT), which binds to the dopamine transporter once administered and allows for visualization of dopaminergic neuron terminals through single-photon emission computed tomography (SPECT) imaging. On the other hand, imaging for VMAT-2, a protein responsible for packaging neurotransmitters like dopamine, serotonin, and norepinephrine into vesicles within presynaptic neurons, was performed only for PD subjects from Australia with the assistance of 18F AV133. The 18F AV13 (a radiolabeled designed ligand) binds to VMAT2 and is synthesized using fluorine-18, a positron-emitting isotope that allows for imaging via positron-emission tomography (PET) for the visualization of changes in dopaminergic function (Please refer to **Supplementary Materials** for the details procedures and descriptions for DAT and VMAT-2 Imaging). On the standard space, with the assistance of a standard striatal template, all images were interpreted as positive or negative for DAT or VMAT-2 deficit based on the intensity and symmetry of radiotracer uptake in the left and right putamen. This process was performed visually by two experienced radiologists specializing in PD. The deficit in DAT or VMAT-2 was recorded and reflected on the product label. Of importance is that, in addition to clinical features of PD, subjects with DAT or VMAT-2 demonstrating dopaminergic deficit were enrolled as the PD cohort. Those clinically considered potential PD but had DAT or VMAT-2 scans without evidence of dopaminergic deficit (SWEDD) were enrolled as the SWEDD cohort. Quantitative data from DAT and VMAT-2 images were obtained from four regions: the caudate (left and right), the posterior putamen (left and right), the anterior putamen (left and right), and the occipital cortex (the reference tissue). Note that the posterior putamen is also referred to as putamen in other sections of this study. The DAT or VMAT-2 count densities extracted from each region were used to calculate the SBr for the striatal regions using the following formula: SBr = (target region/reference) - 1, where the DAT count densities of the occipital cortex were used as the reference.

### 2.5 Data preprocessing

Data files were of 462 GB (total size). We used four NVIDIA Geforce RTX 3090 GPU memories each with 24 GB (RAM) to leverage their memories and high-performance computing capability (with Cuda v12). The data were organized in our server, offering 16T (storage space). We created an environment for R v.4.3.1 for the purpose of data organization and statistical analyses. The virtual environment with Python 3.10 was also built for subsequent data processing, including clustering, network construction, and machine learning model fitting.

### 2.6 Statistical analyses (ANOVA and Chi-square)

First, we computed the percentage of sample size for each group in the cohort. Thereafter, we conducted Chi-Square (χ2) and ANOVA tests to determine specific features (including particular genes) associated with healthy status. The computations of the tests were based on presumptions that specific genes might be involved in the neurobiology or pathogenesis of Parkinson’s disease, with the further hypothesis that some genes might accelerate the onset of this disorder more than others. The significance level was set to P < 0.05, implying that the null hypothesis (H0) was rejected when P < 0.05, suggesting that there is enough evidence to accept the alternative hypothesis (H1), which supports the existence of a strong relationship or association between the variables being evaluated. Of note is that χ2 is a non-parametric test, examining the differences between observed and expected values to determine potential associations between categorical variables (here we included variables such as sex and APOE genotype). We specifically used χ2 test algorithm implemented in the chisq.test () function in R and further applied Fisher’s exact test to enhance the validity of the results where the chi-square approximation might have been inaccurate. With regard to ANOVA, we specifically used it to determine whether the means of multiple independent groups (i.e., PD, SWEDD, and HC) were different. This was done by leveraging the analysis of variances between groups and within groups. Considering that factors such as age might affect the results, we introduced ANCOVA, typically implemented in R as ANOVA Type III, to account for the effect of age when assessing the differences across groups and their possible interactions.

### 2.7 Clustering and machine learning

#### 2.7.1 Dimensionality reduction

From observation, we noticed that some features from brain structures were highly correlated, which might affect the K-means clustering algorithm. Thus for simplicity and for highlighting the features that are highly distinct, we recruited the dimensionality reduction strategy. Here we used Principal Component Analysis (PCA) to identify the highly distinct dimensions of the features that would aid the K-means algorithm to perform fairly better. Components were generated and each set of components was later tested in the K-means algorithm to see how many clusters would be generated (groups of similar patterns). In fact, we capitalized on the PCA class and its method (fit_transform) given by sklearn.decomposition module to generate the PCA components of our interest. Given that PCA decomposition works well on the standardized data, we accordingly standardized our data (using z-score, i.e., subtracting the sample mean from each feature point and scaling to unit σ) prior to PCA decomposition using the method called fit_transform implemented in StandardScaler class in the sklearn.preprocessing module. We then run the PCA algorithm four times to obtain four sets of PCA components(ζ1 = 2, ζ2 = 3, ζ3 = 4, and ζ4 = 5) that would be later tested to find out which set of PCA components would yield better clustering of the data in K-means clustering.

#### 2.7.2 K-means clustering

Here, the goal was to identify subjects within the PD and SWEDD groups that exhibit similar patterns and compare them with others that exhibit different patterns despite all being in the PD or SWEDD groups. The identification of subjects that display similar patterns against others that display different patterns could aid in the discovery of the different manifestations of Parkinson’s disease or reveal other dimensions of Parkinson’s disease subtypes based on these patterns originating from brain structures. To this end, we employed the K-means clustering method, which has been widely used in earlier studies. Specifically, we first grouped all subjects (whole sample, i.e., HC+PD+SWEDD) and subject their structural information of the brain into the K-means algorithm. These techniques not only help avoid biases in the data but also enable us to understand the individual variations of the features and severity of the brain changes. It is possible that despite the majority of people being healthy or unnoticed with illness, they are likely to undergo changes that may be similar to those seen within PD or SWEDD. In a similar fashion, in some of those with PD, or SWEDD, there may be structures that remain intact despite disease (at least sharing similar patterns with those who are healthy). The K-means clustering algorithm is the best candidate for answering these questions and understanding such phenomena. We thus harnessed the K-means capability implemented in sklearn.cluster module. We performed K-means on the sets of PCA components described in section 2.3.2. We first initialized the centroid seeds and ran the K-means algorithm. For each run, the K-means algorithm performed 500 iterations to reach its final decision. We also allowed the K-means to run 5000 times with different centroid seeds to ensure the reproducibility of the results, while the random state was constant. This process was repeated for each set of PCA components drawn from the primary features. In the process of assigning each point to a particular cluster, the Euclidean distance was computed and points with min Euclidean distance from each other were considered to belong to the same cluster. Three criteria were used for the evaluation of an optimal number of clusters, i.e., Silhouette, Calinski-Harabasz, and Davies-Bouldin-based criteria. Each method employed in these criteria contributed to the average optimal number, which was set to be the final optimal cluster number for each set of the PCA components subjected to the K-means algorithm.

#### 2.7.3 Hierarchical clustering

We also explored the capability of a hierarchical clustering model for data clustering in our study. We gained two benefits from this model. First, under this model, we did not need to initialize the centroids before conducting the clustering, which is typical in the K-means algorithm. Second, we learn a separate learning process of the features using a recursive process, which is likely to end up in learning different aspects of information that are unlikely to be captured by the K-means algorithm.

Similar to the K-means algorithm, we trained two separate sets of features into the hierarchical clustering algorithm. The first set of features contained the structural information of the six brain features, and the second set of features contained the structural information of the four brain structures, leaving away the information of the brain structures that seemed to be less distinct from other brain structures (highly correlated). Since our goal is to determine and cluster the observations that are highly associated, we thus transposed our initial matrices of data (N×M) for both sets of data (i.e., 4-dimension and 6-dimension structural features) to obtain M×N data, where N is the number of observations (subject number in this case), and M is the number of features. Next, we performed the Pearson correlation algorithm to obtain the correlation coefficients (in N×N matrices), signifying the extent of similarity in features across the subjects.

We next leveraged the agglomerative clustering approach to our new N×N matrices for data clustering. We first computed the proximity (similarity) matrix using Euclidean distance, after initializing each data point as a cluster. We then used the “ward” linkage strategy to merge the clusters based on the metric of similarity between the clusters. This was followed by updating the similarity matrix after each time the two clusters merged. We repeated the process until a single cluster remained.

The complete algorithm for Ward’s variance minimization strategy is given by


$$d\left(u,v\right)=\;\sqrt{\frac{\left|u\right|+\left|s\right|}{T}d{\left(v,s\right)}^{2}+\frac{\left|v\right|+\left|t\right|}{T}\;d{\left(v,t\right)}^{2}-\frac{\left|v\right|}{T}d{\left(s,t\right)}^{2}}$$

Where ‘u’ is the new cluster consisting of ‘s’ and ‘t’, and ‘v’ is the unused cluster in the forest. ‘t’ is equal to the sum of the number of elements in clusters ‘v’, ‘s’, and ‘t’.

Each iterative update combines clusters ‘s’ and ‘t’ from n samples that are similar to each other, removes them from the cluster group, and uses a new cluster ‘u’ instead. The decision to determine the number of clusters was based on the cut-off (threshold) we set for the Euclidean distance, which determined how many clusters were formed under such a threshold. For that case, we set two thresholds for our data, each threshold for a separate clustered-N×N-correlation matrix depending on the nature of an original number of brain structures used to construct the matrix. The full process of hierarchical clustering is automated by the linkage function offered by the hierarchy module (scipy.cluster.hierarchy class) in the scipy package in Python. The function returns a (N-1) × 4 matrix that contains information on the clustering tree. The information regarding the first and the second clusters that are merged are encoded in the first two columns, the Euclidean distance for merging the two clusters is encoded in the third column, while the fourth column encodes the number of elements present in this newly formed cluster.

#### 2.7.4 Machine learning models

We used three ML models (SVM, Logistic Regression, and Random Forests) to investigate the diagnostic powers of the brain features for distinguishing the SWEDD and PD from HC controls, and for distinguishing different sets of clusters associated with SWEDD, PD, or PD subtypes from those associated with HC. For the first goal, we organized the data in such a way the features from brain structures can be used to distinguish PD and SWEDD from the controls. Herein we initially utilized the Logistic Regression Model provided by the linear model module of sklearn package. We split the data, 80% for training and 20% for testing. We trained the Logistic Regression with the default parameters and later tested it; the model performance was evaluated based on the estimates of AUC and Accuracy. We repeated these steps for SVM and Random Forest models. The Random Forest model was initialized with 100 decision trees while the SVM model was initialized with linear kernels for hyperplane estimation.

During the learning process of the models, we identified the features that have higher predicting power for differentiating targeted classes using the feature importance identification function. For the second goal, i.e., differentiating the sets of clusters (networks) associated with SWEDD, PD, and PD subtypes from those associated with HC, we trained the models in such a way that features from the brain structures targeted predicting the clusters obtained initially from K-means algorithm. How accurately the models could learn the brain feature to predict the same patterns of the clusters was tested by the AUC and accuracy of the models. There are two advantages to doing this type of learning: first, this learning process enables us to assess how close the clustering algorithm is to other ML models. Second, the approach enables us to reveal the diagnostic power of brain structures into understanding different patterns of the networks associated with the majority of individuals with SWEDD, PD, and potential PD subtypes, which may be hardly identified in other approaches.

## 3 Results

### 3.1 Datasets and demographics

**Figure 1:**
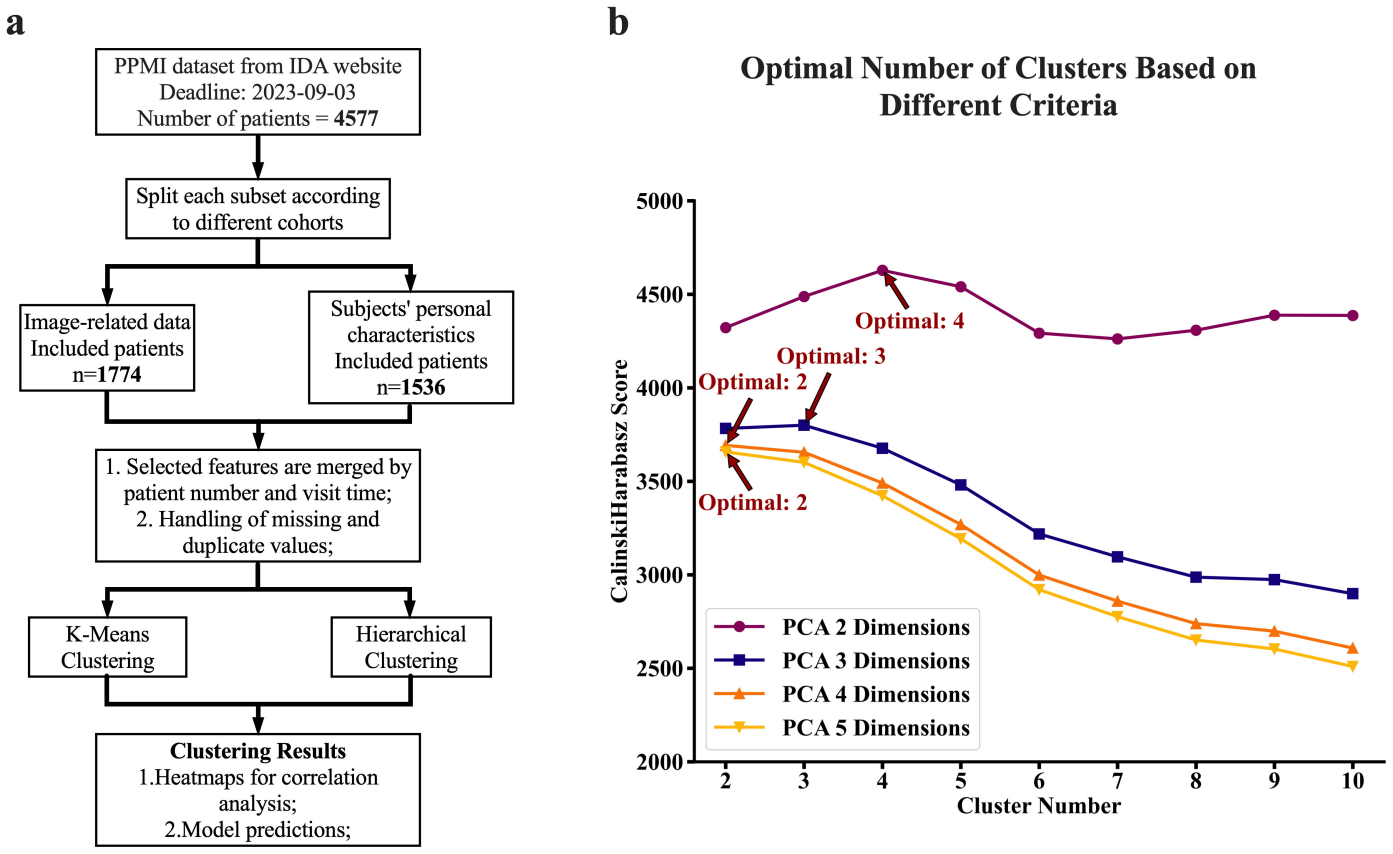
Experimental flow chart and clustering criteria. **(A)** Flow chart of the experimental design. We first screened the PPMI dataset from the IDA database and split the data into different cohort categories. We next obtained records of volume measurements of the brain for each subject, which were primarily obtained from MRI scans. We further collected their corresponding demographic characteristics and blood test results including DNA extracted sequences. Next, we performed chi-square and ANOVA analyses for relationship assessment among the key factors (variables). Furthermore, the clustering analyses (both K-means and Hierarchical) were performed for the identification of patterns associated with disease against those associated with healthy status. Lastly, we conducted further experiments to determine the diagnostic powers of these features for Parkinson's disease and subtypes using machine learning models. **(B)** The computed optimal number of clusters is based on a different set of PCAs (Principal Component Analysis). Calinski Harabasz's method determined the optimal number of clusters on different sets of PCA numbers, reflecting different dimensions of features. We obtained these sets of PCA by subjecting the raw six-dimension features from the brain to the PCA analyses. The reduced features (PCAs) after PCA analyses were next utilized for clustering analyses. The plot **(B)** was a cluster number versus Calinski Harabasz score, where for each set of clustering, the highest Calinski Harabasz score was regarded as the optimal number of clusters per Calinski Harabasz criteria.

The *PPMI* dataset consisted of data from *4577* participants, obtained from *50* different sites. The key data in the cohort included prodromal (from *2,612* participants), *PD* (from *1,547* participants), *HC* (from *328* participants), *SWEDD* (from *81* participants), and *AV133* (from *9* participants, with newly developed F-AV133 biomarker of *PD*) data. The Prodromal cohort was made of data of individuals who were at risk of developing Parkinson’s disease based on clinical features, genetic variants, or other biomarkers (e.g., proteome). Please refer to **Supplementary Table S7** for more information regarding the prodromal cohort. Subjects in the *PD* cohort either had early-stage, untreated sporadic Parkinson’s disease or Parkinson’s disease caused by a genetic variant. The HC cohort comprised subjects who did not have any neurological disorders and had no first-degree relatives with Parkinson’s disease. Meanwhile, the *SWEDD* cohort involved the subjects who were clinically diagnosed with Parkinson’s disease but with normal results on visual inspection of DAT SPECT scans. Of all participants, *2184(47.72%)* were female, *2391(52.24%)* were male, while 2 individuals were with undeclared gender. After critical data screening and processing (see **Figure 1A**), only a subset of this large cohort was used for the subsequent analyses. The demographics of the screened data are reported in **Table 1** (Also, see **Supplementary Table S8** for an additional summary (in means and SD) of the demographics). Only three groups (i.e., *PD, HC, and SWEDD*) were eventually left for further analyses.

**Table 1:** Descriptive statistics and tests for medical variables in PPMI data.

	PD	HC	SWEDD	
	N=2037	N=222	N=137	
Factors	No. (%)	No. (%)	No. (%)	*p*-Value
SEX				
Female	759 (37.3%)	71 (32.0%)	55 (40.1%)	0.219
Male	1278 (62.7%)	151 (68.0%)	82 (59.9%)	
HANDED				
Right	1761 (86.5%)	179 (80.6%)	115 (83.9%)	**0.034**
Left	217 (10.6%)	28 (12.6%)	16 (11.7%)	
Mixed	59 (2.9%)	15 (6.8%)	6 (4.4%)	
RAWHITE				
No	84 (4.1%)	12 (5.4%)	5 (3.6%)	0.628
Yes	1953 (95.9%)	210 (94.6%)	132 (96.4%)	
APOE				
E2/E2	18 (0.9%)	4 (1.8%)	4 (2.9%)	**<0.001**
E2/E3	257 (12.6%)	21 (9.5%)	18 (13.1%)	
E2/E4	33 (1.6%)	9 (4.1%)	10 (7.3%)	
E3/E3	1287 (63.2%)	139 (62.6%)	63 (46.0%)	
E3/E4	408 (20.0%)	42 (18.9%)	37 (27.0%)	
E4/E4	34 (1.7%)	7 (3.1%)	5 (3.7%)	
LRRK2_POS				
Normal	117 (5.8%)	11 (5.0%)	2 (1.5%)	0.184
Variant	1918 (94.2%)	211 (95.0%)	135 (98.5%)	
ONLINE_ENROLL				
No	1838 (90.2%)	191 (86.0%)	131 (95.6%)	**0.012**
Yes	199 (9.8%)	31 (14.0%)	6 (4.4%)	
Median (IQR)				
AGE_AT_VISIT	63.9 (56.6-70.5)	61.45 (54.6-68.675)	63.5 (52.6-68.3)	**<0.001**
CAUDATE_L	1.7 (1.32-2.09)	2.915 (2.52-3.35)	2.7 (2.3-3.21)	**<0.001**
CAUDATE_R	1.67 (1.3-2.1)	2.825 (2.51-3.278)	2.77 (2.36-3.09)	**<0.001**
PUTAMEN_L	0.62 (0.48-0.83)	2.05 (1.765-2.48)	1.94 (1.57-2.37)	**<0.001**
PUTAMEN_R	0.63 (0.48-0.84)	2.08 (1.723-2.52)	1.96 (1.63-2.31)	**<0.001**
PUTAMEN_L_ANT	1.05 (0.81-1.34)	2.49 (2.19-2.928)	2.31 (1.99-2.76)	**<0.001**
PUTAMEN_R_ANT	1.06 (0.83-1.37)	2.52 (2.15-2.978)	2.4 (2.02-2.85)	**<0.001**
RNASEQ_VIS	4 (3-5)	5 (4-5)	4 (3-4)	0.142
EDUCYRS	16 (14-18)	16 (14-18)	15 (12-17)	0.526

PD, Parkinson disease; HC, healthy control; SWEDD, scan without dopaminergic deficit; HANDED, handedness; RAWHITE, the white race; APOE, apolipoprotein E; LRRK2_POS, leucine-rich repeat kinase 2; CAUDATE_L, striatal binding ratio of the left caudate small brain region of interest; CAUDATE_R, striatal binding ratio of the right caudate small brain region of interest; PUTAMEN_L, striatal binding ratio of the left putamen small brain region of interest; PUTAMEN_R, striatal binding ratio of the right putamen small brain region of interest; PUTAMEN_L_ANT, striatal binding ratio of the left anterior putamen small brain region of interest; PUTAMEN_R_ANT, striatal binding ratio of the right anterior putamen small brain region of interest; RNASEQ_VIS, count of RNA-sequencing visits with data; EDUCYRS, number of years of education. The upper part includes categorical variables and the others are continuous variables. The bold values in **Table 1** represent the P-values that are statistically significant at P < 0.05.

### 3.2 Genotypes and Parkinson’s disease

The results of the association between *APOE* genotype and Parkinson’s disease are summarized in **Table 1**. Our analyses show that there is significant association between APOE genotype and healthy status (χ2 = 44.818, DF =10, p < 2.345×10^-06^). The χ2 *post-hoc* analyses show that there is an almost equal distribution of *APOE* genotypes across *HC, SWEDD, and PD* for ϵ2/ϵ2, ϵ2/ϵ3, ϵ3/ϵ4, and ϵ4/ϵ4 *APOE* genotypes (p > 0.05) (see **Supplementary Table S3**). However, there is significant evidence to suggest an association between Parkinson’s disease and the *APOE* genotype, such that *PD* is highly associated with *APOE* ϵ2/ϵ4 (p < 0.0004), and ϵ3/ϵ3 (p < 0.0011).

### 3.3 ANCOVA analyses and group differences

**Figure 2:**
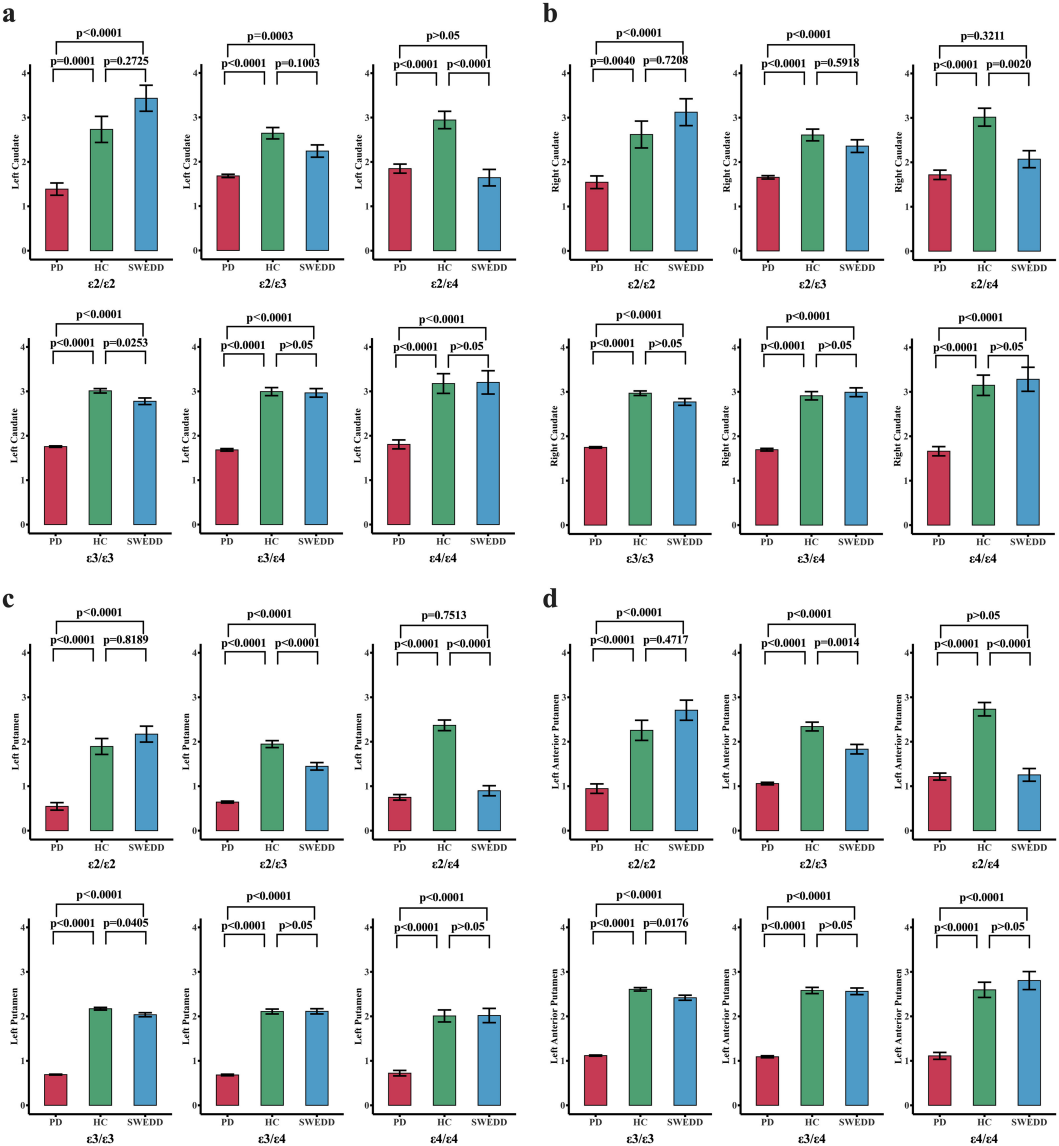
Changes in dopamine transporter striatal binding ratio ((DAT SBr) across different groups stratified by APOE genotype. The red, green, and blue bar plots represent the PD, HC, and SWEDD groups, respectively, across six APOEs. The three groups—PD, HC, and SWEDD—were compared for DAT SBr deficit levels across the six APOEs using estimated marginal means adjusted for APOE genotype levels. For each pair of the groups compared for each APOE genotype, the p-values adjusted for multiple comparisons (Bonferroni correction) are reported. A p-value set at p < 0.05 is considered significant. **(A)** Comparisons of DAT SBr across groups for different APOE genotypes in the left hemispheric caudate nucleus. **(B)** Shows the DAT SBR in the right hemispheric caudate nucleus. The DAT SBr levels for the left putamen and the left anterior putamen are also reported in **(C, D)**, respectively. Generally, these findings demonstrate that subjects with PD exhibit a significant reduction in DAT SBr compared to healthy controls (HC) across all six levels of the APOE genotype. Subjects with SWEDD also exhibited a significant DAT SBr reduction compared to healthy controls, especially for those with the ε3/ε4 APOE genotype. The DAT deficit level was higher in subjects with PD compared to those with SWEDD at all levels of the APOE genotype, except for ε2/ε4 carriers.

**Table 1** also offers a summary of the measurements of DAT SBr for different brain regions across the groups (unadjusted marginal means). Our early evidence from the analysis (prior to *ANCOVA*) indicate that *PD* had relatively the lowest DAT SBr (Example: left caudate; *μ* =1.7; range: 1.32-2.09), followed by *SWEDD* (*μ* =2.7; range: 2.3-3.21), while *HC* had relatively the largest DAT SBr compared to other groups (*μ* =2.9; range: 2.52-3.35). This pattern is observed for both left and right hemispherical regions of caudate and putamen (see **Table 1** for further details). Confirming these preliminary observations, *ANCOVA* analyses (adjusted for age and *APOE* genotype) indicated that the DAT SBr in these regions were significantly different across the three groups (p < 0.001), with subjects with Parkinson’s disease demonstrating highly reduced DAT SBr in the regions (see **Supplementary Table S1**). **Figure 2** elucidates these findings across different levels of *APOE* genotypes, showing that subjects with *PD* exhibit significantly reduced DAT SBr compared to healthy controls, who demonstrate higher DAT SBr across all six levels of the *APOE* genotype (see **Supplementary Figure S1** for more details). We also observed that subjects with *PD* demonstrated a significant DAT deficit compared to subjects with *SWEDD* at all levels of the *APOE* genotype, except for those who were ϵ2/ϵ4 carriers. The findings also show that subjects with *SWEDD* exhibit a significant DAT SBr reduction compared to healthy controls, especially for subjects with the ϵ3/ϵ4 *APOE* genotype.

### 3.4 Clustering analyses

**Figure 3:**
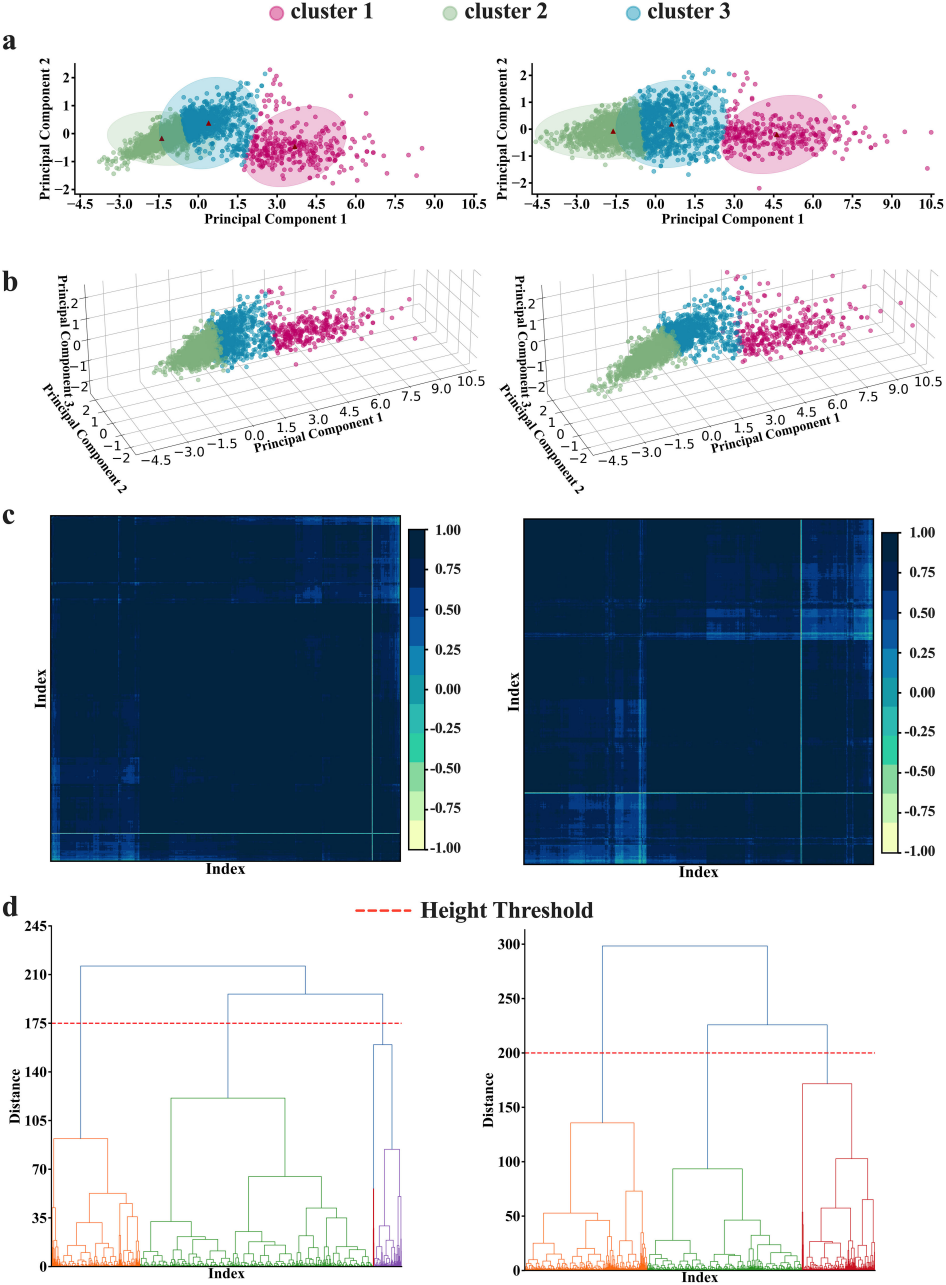
K-means and Hierarchical clustering results. **(A, B)** K-means results in **(A)** 2-dimension and **(B)** 3-dimension representations. The K-means analyses depict 3 as an optimal number of clusters, obtained by averaging the optimal numbers of the three clustering criteria [Silhouette (k=2), Calinski Harabasz (k=4), and Davies Bouldin (k=2)]. **(C, D)** The hierarchical analysis results. The analyses also depict 3 as the optimal number of clusters at the threshold of 175 to 200(h=175 for four features analyses; and h=200 for six features analyses) as illustrated in both **(C)** matrix and **(D)** dendrogram representations. Note the left panel and the right panel present the results conducted with four features and six features, respectively.

Clustering using the K-means algorithm, followed by adjustments based on criteria from the *elbow* method, *Silhouette Coefficient, Calinski-Harabasz index, and Davies-Bouldin index*, resulted in three distinct clusters, suggesting that brain features in cohort can be viewed as a set of three distinct patterns of features. The number of clusters generated by K-means was independent of number of *PCA* components fed into the algorithm as long as the *PCA* components were generated from 6 regions of the brain. However, when feeding the algorithm with *PCA* components generated from four features of the brain, the resulting optimal number of clusters varies depending on the number of *PCA* components. Two *PCA* components result in four optimal clusters, while three *PCA* components result in three optimal clusters (See **Figure 1B**). **Figure 3** provides a visualization of the three consistently generated clusters after feeding the algorithm with *PCA* components derived from brain features. On the other hand, applying hierarchical clustering to our data, we obtained three optimal clusters at thresholds of *175* and 200 (see **Figure 3D**). The threshold of *175* was for clusters derived from four brain features, while the threshold of *200* was for clusters derived from six brain features. Of *2396* participants *(PD=2037, HC=222, SWEDD=137)*, the first two clusters accounted for *47%* and *39%* of the entire dataset, while the third cluster accounted for *14%* of the entire dataset.

### 3.5 PCA components and brain features

We performed correlation analysis to identify which patient’s features correlate most strongly with PCI. In the analysis involving six features (see **Supplementary Table S4**), we learn that the PC1 is most highly correlated with putamen-anterior features, while in the analyses involving 4 brain features, PC1 is fairly correlated with features from both caudate nucleus and putamen (all with r > 0.92), see **Supplementary Table S4**), although the degree of correlation strength differs across clusters. Supporting this observation are the results from the analysis of feature contribution to each principal component. Wherein, we observed that features from both the caudate nucleus and putamen in both hemispheres contribute almost equally to PC1 and PC2. The only difference is that PC1 has a positive relationship with these brain features, while PC2 is formed with both positive and negative combinations of the features (see **Supplementary Table S5** for feature contribution).

### 3.6 Correlation analyses and patterns of networks

**Figure 4:**
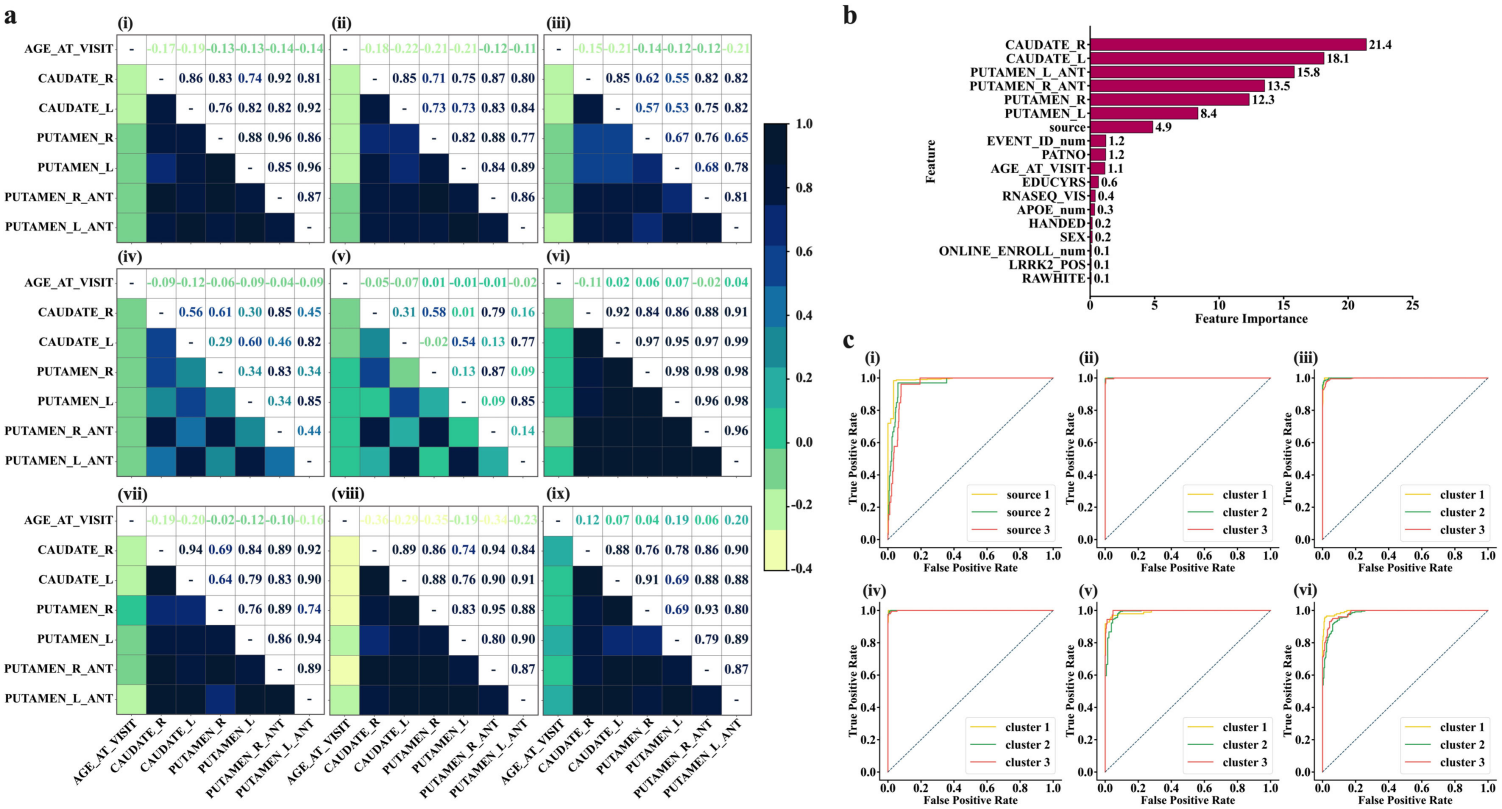
Characterization of state networks and diagnostic performance of ML models for distinguishing SWEDD and PD from HCs. **(A)** Characterization of HC, SWEDD, and PD using state networks. Top panel-left (Img.1); the global characterization of the features’ network of the whole cohort (HC+SWEDD+PD). The overall state network of the whole sample is typified by highly dense and strong connectivity (high level of correlations), purportedly indicating strengthened structural integrity. This highly dense and strong connectivity appears to be highly contributed by HC, as demonstrated by HC’s state network (top panel-middle (Img.2); HCin_K-cluster1 = 84.23% of the HC sample, N = 187 of 222). The SWEDD subjects (top panel-right (Img.3); SWEDDin_K-cluster1 = 73.72% of the SWEDD sample, N = 101 of 137)) are characterized by strong connections but with significant reduction of the putamen and caudate structural connectivity compared to HCs. Middle panel–left (Img.4); state network characterization of subjects with PD in K-cluster2 (PDin_cluster2 = 55.28% of the PD sample constituting cluster 2, N = 1126 of 2037). Subjects with PD in K-cluster2 exhibit a sparse network. Middle panel-middle (Img.5); the PD subjects in K-cluster3 (PDin_cluster3 = 42.56% of the PD sample, N = 867 of 2037) typified by a more sparse network compared to PD subjects in K-cluster3. Img.6 to 9 display the characterization of the state networks resulted after the hierarchical clustering. Bottom panel-right (Img.9); healthy subjects (HC) in Hirar-cluster3 characterized by intensified level of connectivity among structures and among structures and age at visit. Those who are SWEDD (Hirar-cluster3, middle panel-left, Img. 6) are typified by the high level of connectivity across brain features and age, but the connectivity with age was slightly lesser compared to HC individuals. Subjects with PD in Hierarchical clustering were mainly in Hirar-cluster1 and Hirar-cluster3. The PD in Hirar-cluster1 (bottom panel-left, Img. 7) shows a slightly lower strength of connectivity in brain structures and between brain structures and age compared to SWEDD subjects. Those PD subjects in Hirar-cluster3 (bottom panel-middle, Img. 8), however, display a network typified with highly reduced structural connectivity with age, although the connections between brain structures themselves appeared to be intact. **(B, C)** performances on Machine Learning algorithms. **(B)**, Features ranked per their contribution to the machine learning models. The features from brain structures play a vital role in the diagnosis of SWEDD, PD, and PD subtypes. **(C)** Diagnostic performances of Machine Learning models for distinguishing SWEDD and PD from HC. **(C)**, top panel-left (Img. 1); diagnostic performance of SVM. **(C)**, top panel-middle (Img. 2); SVM performance in distinguishing different K-mean-derived state networks describing PD subtypes. **(C)**, top panel-right (Img. 3); Random Forest performance for distinguishing K-means derived state Network patterns. c, bottom panel-left(Img. 4); Logistic Regression Performance in distinguishing K-mean derived state network patterns. c, bottom panel-middle (Img. 5); SVM performance in distinguishing 4-feature-based Hierarchical-derived state network patterns. c, bottom panel-right(Img. 6); SVM performance in distinguishing 6-feature-based Hierarchical-derived state network patterns.

Following identifying the optimal number of clusters using K-mean algorithm, we performed a Pearson correlation on the data for participants within the same clusters to observe the patterns of features that each cluster would disclose. The heatmap representation shows that cluster 1 is characterized by brain features that exhibit highly dense and strong connectivity in the network (**Figure 4A**, top panel, left). The highly dense and strong connectivity in the global network of features in cluster *1* (constituting data from *HC+SWEDD+PD*), as depicted by correlation coefficients, appears to be significantly contributed by *HC* (*187 of 222 (84.23%) HCs* contribute to this cluster). This can be clearly observed through HC’s network (**Figure 4A**, top panel, middle) obtained by using data from only *HC* participants that belong to cluster 1. The heat map representation (**Figure 4A**, top panel, right) shows that subjects in the *SWEDD* group that belong to cluster 1 are characterized by strong connectivity within the structural sub-regions, with a particularly significant reduction in putamen and caudate structural connectivity compared to *HCs* in the same cluster. While both clusters—cluster 2 and cluster 3— are dominated by data from Parkinson’s disease (*1126* out of *2037 (55.28%)* for cluster 2 and *867* out of *2037 (42.56%)* for cluster 3), heat map representations reveal distinct structural network patterns in the global network for each cluster. Subjects with *PD* in cluster 2 exhibited a sparse network, suggesting a moderate loss of structural integrity (**Figure 4A**, middle panel, left, also see **Supplementary Table S6** for sparsity), while subjects with *PD* in cluster 3 demonstrated an even more intensive sparsity than those in cluster 2 (**Figure 4A**, middle panel, middle), likely reflecting a more advanced loss of structural integrity due to the advanced level of *PD* neuropathology. We also observed that these PD subtypes, described by the two network states of distinctive patterns, were independent of APOE genotype (χ2 tests, P > 0.05).

Using information from hierarchical clustering, we observed that subjects in HC classified into cluster 3 were characterized by intensified connectivity among brain structures and age at visit (**Figure 4A**, bottom panel, right). Subjects with *SWEDD* (**Figure 4A**, middle panel, right), on the other hand, demonstrated the same level of strong connectivity across brain structures but with a slight decrease in connectivity with age at visit compared to *HC* subjects of the same cluster. Subjects with *PD* were highly distributed within cluster 1 and cluster 2, as determined by hierarchical clustering (The order of clusters should not cause confusion; the focus should be on the patterns of features demonstrated by each group within and across clusters). Subjects with *PD* in cluster 1 were characterized by slightly lower connectivity strength in brain structures and between brain structures and age at visit compared to subjects in either *SWEDD* or *HC* (see **Figure 4A**, bottom panel, left). *PD* subjects in cluster 2, however, displayed a network typified by a more reduced connectivity with age, although the structural connectivity across the brain regions remained relatively intact (see **Figure 4A**, bottom panel, middle).

### 3.7 DAT and total grey matter levels in PD subtypes/clusters

Further characterization of the two clusters of PD, cluster 2 and cluster 3, reveals that the PD subjects in cluster 3, characterized by a highly sparse brain DAT network, had lower DAT levels (severe DAT deficit, as quantified by DAT SBr) compared to PD subjects in cluster 2 in almost all six striatal regions assessed across APOE genotypes (see **Supplementary Figure S2**). Similarly, the PD subjects in cluster 3, particularly those with ϵ3/ϵ3, ϵ3/ϵ4, ϵ4/ϵ4 genotypes, appeared to have significantly lower total grey matter volume compared to PD subjects in cluster 2 (see **Supplementary Figure S3**). The results support the potential role of the two identified clusters as representations of moderate and more advanced levels of PD neuropathology.

### 3.8 Machine learning and diagnostic performance

To determine whether the features extracted from brain structures or those derived from them could serve as reliable biomarkers of early, intermediate, and late stages of PD, we employed machine learning models (SVM, Logistic Regression, and Random Forest) and evaluated the diagnostic performance of each model. Our results showed that 6 features of the brain structures can serve as important predictors of PD subtypes (**Figure 4B**), with rankings of *21.4%, 18.1%, 15.8%, 13.5%, 12.3%, and 8.4%*, for *CAUDATE_R, CAUDATE_L, PUTAMEN_L-ANT, PUTAMEN_R-ANT, PUTAMEN_R, and PUTAMEN_L*, respectively.

Upon comparison, we observed that of the three *ML* models, the *SVM* had the highest predictive performance with an AUC of *0.97*, which is only 0.01 lesser than if all features (excluding source) were included in the prediction (**Figures 4B, C**). The mean accuracy and F1 scores of this model were as high as *93.3%* and *91.45%*, respectively.

On the other hand, when predicting the different states (clusters generated by K-means algorithm) at which the *PD* exists, it was found that *SVM* outperformed the others by a small margin (including, Random Forest and Logistic Regression, see **Supplementary Table S2**, also see **Figures 4B, C**). The *SVM* fine-tuned with a linear kernel showed an AUC of 0.99996, with an accuracy of 99.38% and an F1 score of 0.9937. Compared to the Linear Kernel-SVM, the *SVM* fine-tuned with the *RBF* kernel showed slightly lower performance, with an AUC of 0.9875 and an F1 score of 0.9875. Predicting the states derived from the hierarchical clustering algorithm, we observed that we could achieve a maximum performance of 0.9949 AUC and an F1 score of 0.9687 using an *SVM* fine-tuned with a linear kernel. Notably, the best *ML* prediction of the states occurred on clusters that underwent redundant feature control (i.e., 4 feature-derived clusters rather than 6 feature-derived clusters).

Of note is that employing one-vs-rest strategy in our learning models to validate how well each model trained on features of the specific cluster can learn to distinguish specific cluster from all others. Our results (also see **Supplementary Table S2**) show that the models trained on this strategy have improved performance compared to the models trained on all data (a global multi-class classifier), suggesting the utility of the clusters in distinguishing PD-subtypes.

## 4 Discussion

In this study, we show that *PD* is associated with APOE ϵ2/ϵ4 and ϵ3/ϵ3 genotypes. Previous studies have reported the involvement of APOE4 allele in exacerbating α-synuclein pathology in PD (12), tau pathology, neuroinflammation, and Aβ clearance disruption in AD (13–15). The APOE4 genotype increases α-synuclein misfolding and aggregation, forming amyloid fibrillary structures that lead to neurodegeneration in PD (6). The misfolding or genetic mutations in α-synuclein gene and the loss of dopaminergic neurons are thought to be the central mechanism in PD. Increased α-synuclein misfolding and pathology in mouse models and humans have been shown to accelerate cognitive decline and worsen neuronal and synaptic loss (12). This phenomenon appears to increase tenfold in the APOE4 genotype in PD (16). Our data showing a significant association between APOE ϵ2/ϵ4 genotype provide further evidence to support these earlier findings.

Our findings also reveal a significant volume reduction in subjects with *PD* compared to subjects with SWEDD or HC. We show that grey matter atrophy of the subcortical structures, especially the putamen and caudate, can predict the trend of *PD* progression from *SWEDD* to an advanced level of *PD*. Gray matter atrophy of subcortical structures, including the basal ganglia, has been extensively studied. Subjects with early-stage PD without dementia reported significant volume reductions in the putamen, nucleus accumbens, and hippocampus, with shape deformations in the putamen. (17). In demented PD, studies have found a more extensive atrophy in additional regions, such as caudate and parahippocampal gyrus (18). Our data showing a significant volume reduction in the caudate and putamen support these early findings and align with other longitudinal studies indicating progressive atrophy in these areas in the early to middle stages of PD (19).

Our findings also show that a particular *APOE* genotype, especially APOE ϵ2/ϵ4, may accelerate brain atrophy regardless of the stage of *PD or* subtypes. Here, we observed that subjects with SWEDD and PD both have reduced DAT SBr, reflecting the reduction of dopaminergic neurons. The DAT SBr for both SWEDD AND PD cohorts was below 2.0 (1.65 for SWEDD and 1.85 for PD). Although there are limited studies that provide a direct link between the APOE ϵ2/ϵ4 genotype or APOE ϵ4 allele and exacerbated gray matter atrophy or loss of dopaminergic neurons in PD, there is already enough evidence from other degenerative disorders, such as Alzheimer’s disease, to underscore this relationship. For example, some studies of Alzheimer’s disease showed a greater rate of hippocampal atrophy and cortical thinning in the presence of APOE ϵ4 allele in both individuals progressing from mild cognitive impairment to AD and those with demented AD (20, 21), highlighting the role of APOE ϵ4 in brain atrophy regardless of the stages of AD. Exacerbated α-synuclein pathology (22, 23), accelerated breakdown of the BBB (24), and faster cognitive decline (22) reported in PD patients with APOE ϵ4 allele may be early evidence of accelerated neurodegeneration seen in our PD patients. Along these studies is a preliminary study on patients with primary progressive aphasia, which reported the APOE ϵ2/ϵ4 genotype as a possible risk factor for the condition (25). A subsequent study on a larger sample from the same research group indicated that the ϵ2/ϵ4 genotype, particularly in women, might represent a genetic factor for primary progressive aphasia, with molecular positive heterosis explaining this association (26).

Meanwhile, our clustering data show the existence of three states for brain networks. The majority of healthy subjects exist in a state characterized by highly dense and strong connectivity, which is expected as healthy individuals have their structural integrity intact. On the other hand, the majority of subjects with *SWEDD* exist in a state characterized by strong connectivity within the structures of the brain but with a significant reduction in putamen and caudate structural connectivity when compared to HCs. At the same time, we found that the majority of subjects with PD exist in two distinct states. The first state is typified by a mildly sparsely connected network, suggesting a moderate loss of grey matter structural integrity. In contrast, PD patients in the second state are characterized by more intensified sparsity in their network, likely signaling a more advanced level of structural integrity loss as PD neuropathology advances. In line with our study is a study by Shakya et al. (27) that used a clustering analysis on symptom onset, motor, and non-motor features of PD to characterize PD subtypes and identified two distinct PD subtypes, severe motor-non-motor subtype (SMNS) and mild motor-non-motor subtype (MMNS) (27). The MMNS subtype primarily includes participants who exhibited symptom onset at a young age (25.4–80.1 years), with milder forms of Parkinson’s symptoms and relatively less volume reduction in the caudate, putamen, and other striatum regions compared to participants in SMNS. In contrast, the SMNS subtype is characterized by participants who experienced symptom onset at an older age (35.6-83 years), with more intense motor and non-motor symptoms and greater volume reduction than MMNS. Taken together these findings cement that PD exhibit significant clinical variability among patients, suggesting the importance of considering different these distinctive subtypes in the developing therapeutic targets.

Using ML algorithms, we have shown that features extracted from brain structures can serve as reliable biomarkers for PD subtypes, with varying diagnostic power. The SVM model demonstrates slightly better performance in diagnosing PD subtypes or distinguishing *PD* or *SWEDD* from *HCs* compared to other models evaluated in this study. We observed that SVM has the potential to predict different states of structural configuration at which PD manifests, with a performance of approximately *100% AUC*, *99.3%* accuracy, and *0.993* F1. Such a level of performance is achieved through an SVM fine-tuned with a linear kernel, and when issues of redundancy have been addressed. ML algorithms have recently been preferred for identifying PD subtypes, predicting disease progression, and improving the differentiation between subjects with PD and healthy controls (28–30). Notably, a study by Shiiba et al. (30) used SVM models on shape features (striatal binding ratios, circularity, major axis length for putamen and caudate regions) to improve the classification performance of PD from HC (30). The authors demonstrated that SVM models effectively captured the distinctive features of PD and statistically improved the segregation ability between HC and PD, with top AUCs of 0.995 for circularity and SBRs, 0.990 for circularity alone, and 0.973 for SBRs alone. Another study focused on identifying patient subtypes and disease progression using an ML algorithm identified three distinct patient subtypes (29). The authors achieved accurate projections of disease after initial diagnosis with an average AUC of 0.92 (95% CI; 0.95 ± 0.01) for the slower progressing group, 0.87 ± 0.03 for moderate progression, and 0.95 ± 0.02 for the fast progression group. Taken together, these findings suggest that the use of ML algorithms provides insights to understand PD heterogeneity and improve efforts for therapeutic targets.

### 4.1 Limitation

Our data also stress that despite the high degree of similarity in the clusters produced by both hierarchical and K-means algorithms, there are still some differences in the patterns disclosed by the two methods, with hierarchical clustering showing more variations in age connectivity in the networks.

## 5 Conclusion

In this study, we conducted extensive analyses of brain data from subjects with PD, provided by the notable PPMI database, and showed that APOE ϵ2/ϵ4 and ϵ3/ϵ3 genotypes, total grey matter volume, and subcortical dopaminergic deficit in striatal structures (caudate and putamen) are significantly associated with PD. We also infer that the absence of DAT deficits in some subjects with potential clinical symptoms of PD may be linked to APOE ϵ3/ϵ3, which appears to be highly associated with those subjects with scans without evidence of DAT deficits (SWEDD). While reporting that APOE ϵ2/ϵ4 may be involved in accelerating brain atrophy or reduction of dopaminergic neurons (as quantified by DAT striatal binding ratio (SBr)) in PD, we also report that subjects with PD exhibit intrinsic heterogeneity, which may stem from genetic factors. We also report that this heterogeneity can be characterized into two distinct network states using ML algorithms, reflecting two potential PD subtypes. These findings contribute to the current literature on the necessity of considering such heterogeneity when developing personalized drugs.

## Data Availability

The original contributions presented in the study are included in the article/**Supplementary Material**. Further inquiries can be directed to the corresponding author.
