# Supplementary material for: Identifying network state-based Parkinson’s disease subtypes using clustering and support vector machine models
Source: Front Psychiatry. 2025 Feb 13;16:1453852. doi: 10.3389/fpsyt.2025.1453852 (PMC11865070; doi:10.3389/fpsyt.2025.1453852)
Supplement: Supplementary file 1 [file DataSheet1.pdf]

Supplementary Materials

2.4 Dopamine Transporter (*DAT*) and Vesicular Monoamine Transporter 2 (*VMAT-2*) Imaging and Quantification

All *PD* subjects underwent dopamine transporter (*DAT*) imaging or vesicular monoamine transporter 2 (*VMAT-2*) imaging to identify if the clinical features of *PD* align with dopaminergic features of the brain. The imaging for *DAT* (i.e., presynaptic protein highly concentrated in the striatum dopaminergic neurons) was performed with the assistance of 123I Ioflupane (also called *FP-CIT*), which binds to the dopamine transporter once administered and allows for visualization of dopaminergic neuron terminals through single-photon emission computed tomography (*SPECT*) imaging. Briefly, subjects were injected with 185 *MBq* of 123I Ioflupane, followed by imaging 4±0.5 hours later for 30-45 minutes. Imaging sites transferred raw data to the central core imaging laboratory for quality control, which involved assessing motion, standard reconstruction, attenuation correction, and quantification. To ensure consistency in data reconstruction for the data loaded into the *HERMES* (Hermes Medical Solutions, Stockholm, Sweden) system, the *SPECT* raw data underwent iterative reconstruction without the use of any filter. *HOSEM*-reconstructed data transfer to *PMOD* (*PMOD* Technologies, Zurich, Switzerland) was conducted to enable further data processing, including attenuation correction (via Chang 0 attenuation correction) and data filtering (using a standard Gaussian 3D 6.0 mm filter). To ensure standard anatomical alignment across scans, the resulting data were normalized to a *SPECT* ioflupane reference template in standard space (Montreal Neurological Institute, *MNI*). The assessment for striatal uptake of 123I ioflupane was conducted within the eight hottest striatal slices. The trans-axial slice with the highest uptake was identified, and the surrounding slices were averaged to generate a single slice.

On the other hand, imaging for *VMAT-2*, a protein responsible for packaging neurotransmitters like dopamine, serotonin, and norepinephrine into vesicles within presynaptic neurons, was performed only for *PD* subjects from Australia with the assistance of *18F AV133*. The *18F AV13* (a radiolabeled designed ligand) binds to *VMAT2* and is synthesized using fluorine-18, a positron-emitting isotope that allows for imaging via positron-emission tomography (*PET*) for the visualization of changes in dopaminergic function. Therefore, briefly the subjects received imaging after being injected with 222 *MBq* of *18F AV133*. The first

imaging occurred 50 minutes post-injection for 10 minutes, followed by a second imaging session 80 minutes post-injection, also for 10 minutes. The resulting *PET* volumes were imported into the *PMOD* system for data processing and analysis. Initially, the data were co-registered to the subject's *MRI*, and the transformations obtained from *MRI*-to-*MNI* normalization were used to warp the *PET* volumes to standard *MNI* space. A standard striatal template delineating the striatal areas of interest was overlaid on the normalized *MRI* volume.

On the standard space, with the assistance of a standard striatal template, all images were interpreted as positive or negative for *DAT* or *VMAT-2* deficit based on the intensity and symmetry of radiotracer uptake in the left and right putamen. This process was performed visually by two experienced radiologists specializing in *PD*. The deficit in *DAT* or *VMAT-2* was recorded and reflected on the product label. Of importance is that, in addition to clinical features of *PD*, subjects with *DAT* or *VMAT-2* demonstrating dopaminergic deficit were enrolled as the *PD* cohort. Those clinically considered potential *PD* but had *DAT* or *VMAT-2* scans without evidence of dopaminergic deficit (*SWEDD*) were enrolled as the *SWEDD* cohort. Quantitative data from *DAT* and *VMAT-2* images were obtained from four regions: the caudate (left and right), the posterior putamen (left and right), the anterior putamen (left and right), and the occipital cortex (the reference tissue). Note that the posterior putamen is also referred to as putamen in other sections of this study. The *DAT* or *VMAT-2* count densities extracted from each region were used to calculate the *SBr* for the striatal regions using the following formula: *SBr* = (target region/reference) - 1, where the *DAT* count densities of the occipital cortex were used as the reference.
