# Supplementary figures and images for: Identifying network state-based Parkinson’s disease subtypes using clustering and support vector machine models
Source: Front Psychiatry. 2025 Feb 13;16:1453852. doi: 10.3389/fpsyt.2025.1453852 (PMC11865070; doi:10.3389/fpsyt.2025.1453852)

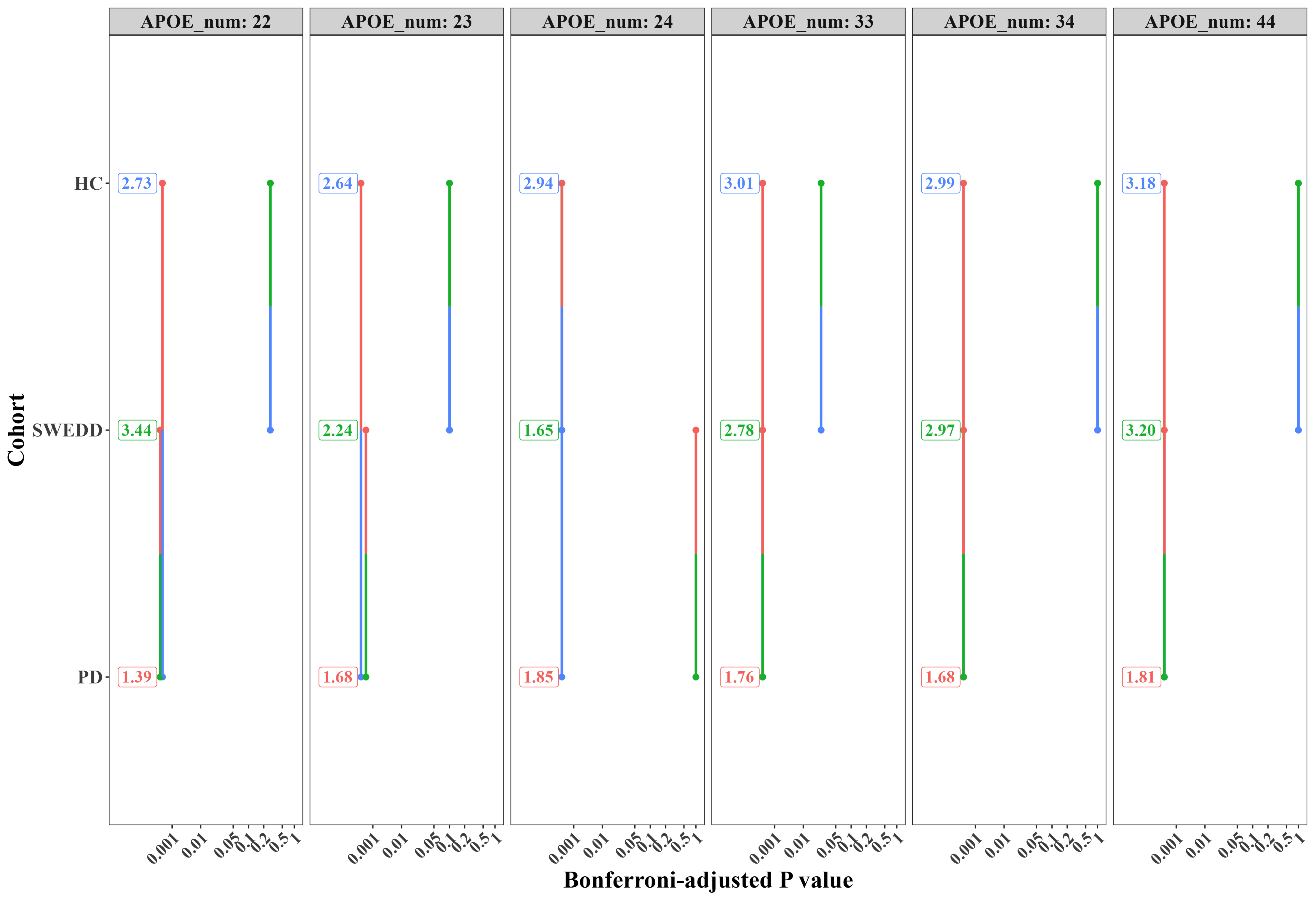

Supplement: Supplementary file 2 [file Image1.tiff]

Supplementary Figure S2. DAT Levels Across Clusters

(i)

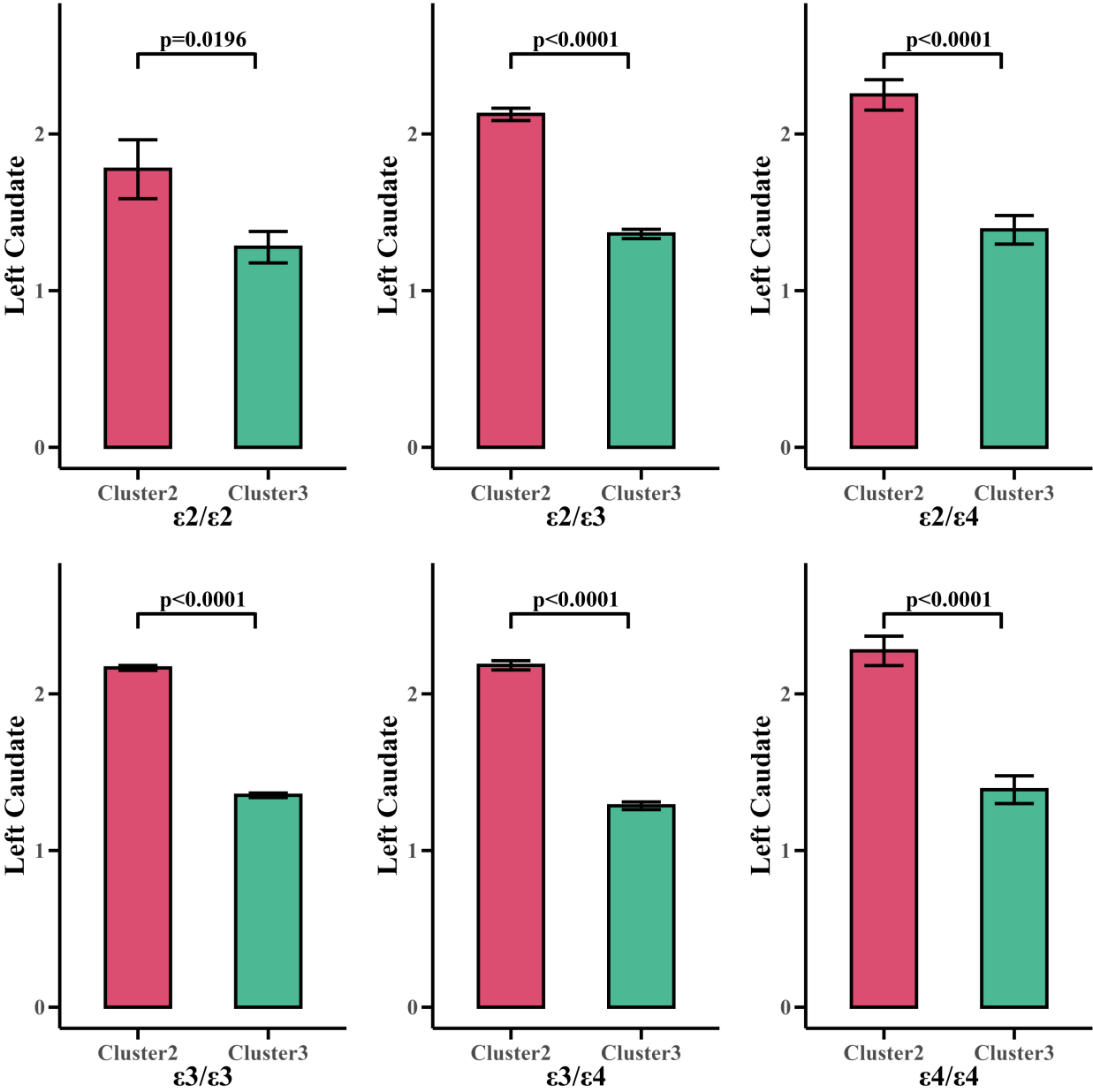

(ii)

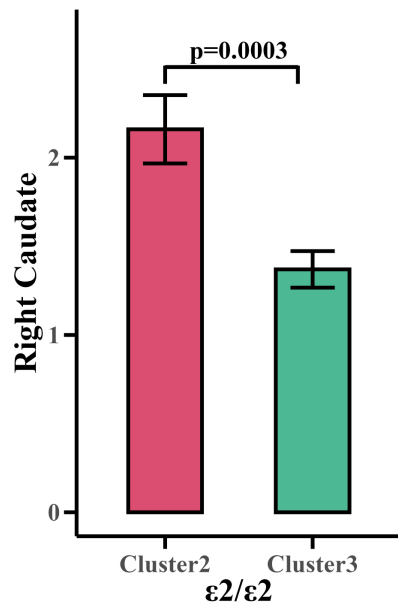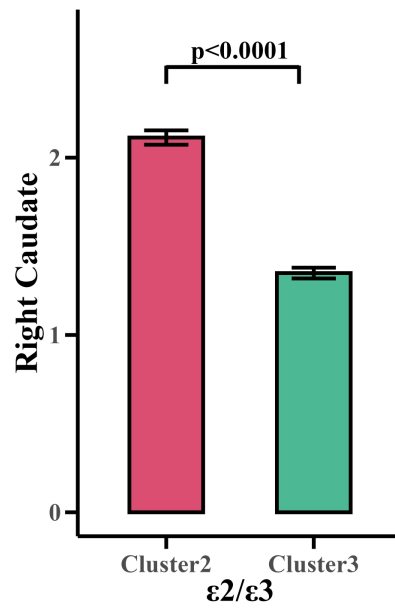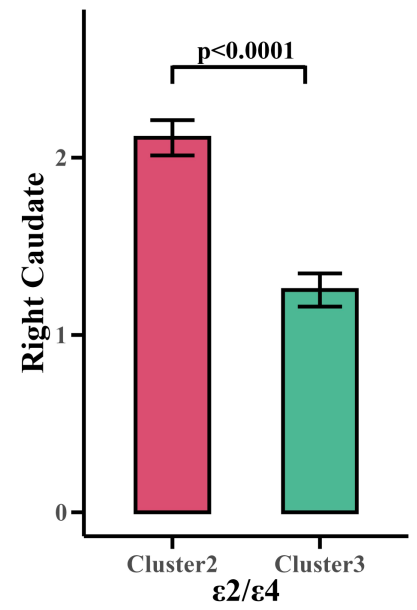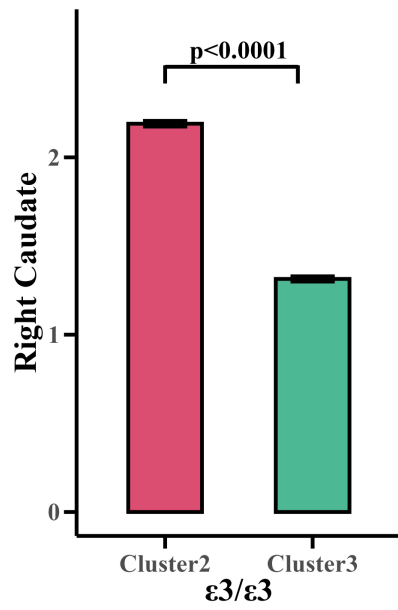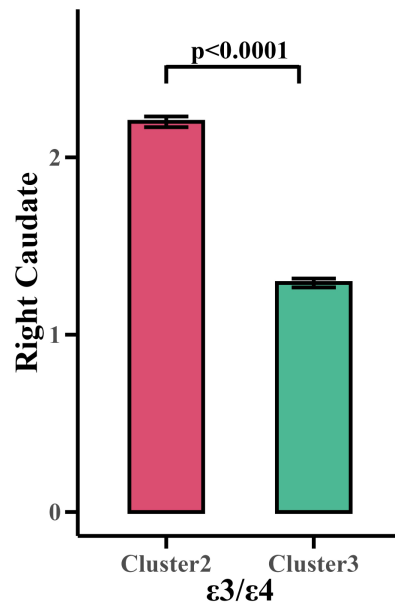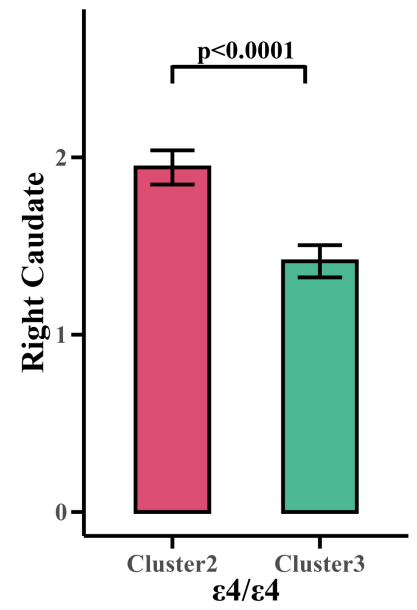

(iii)

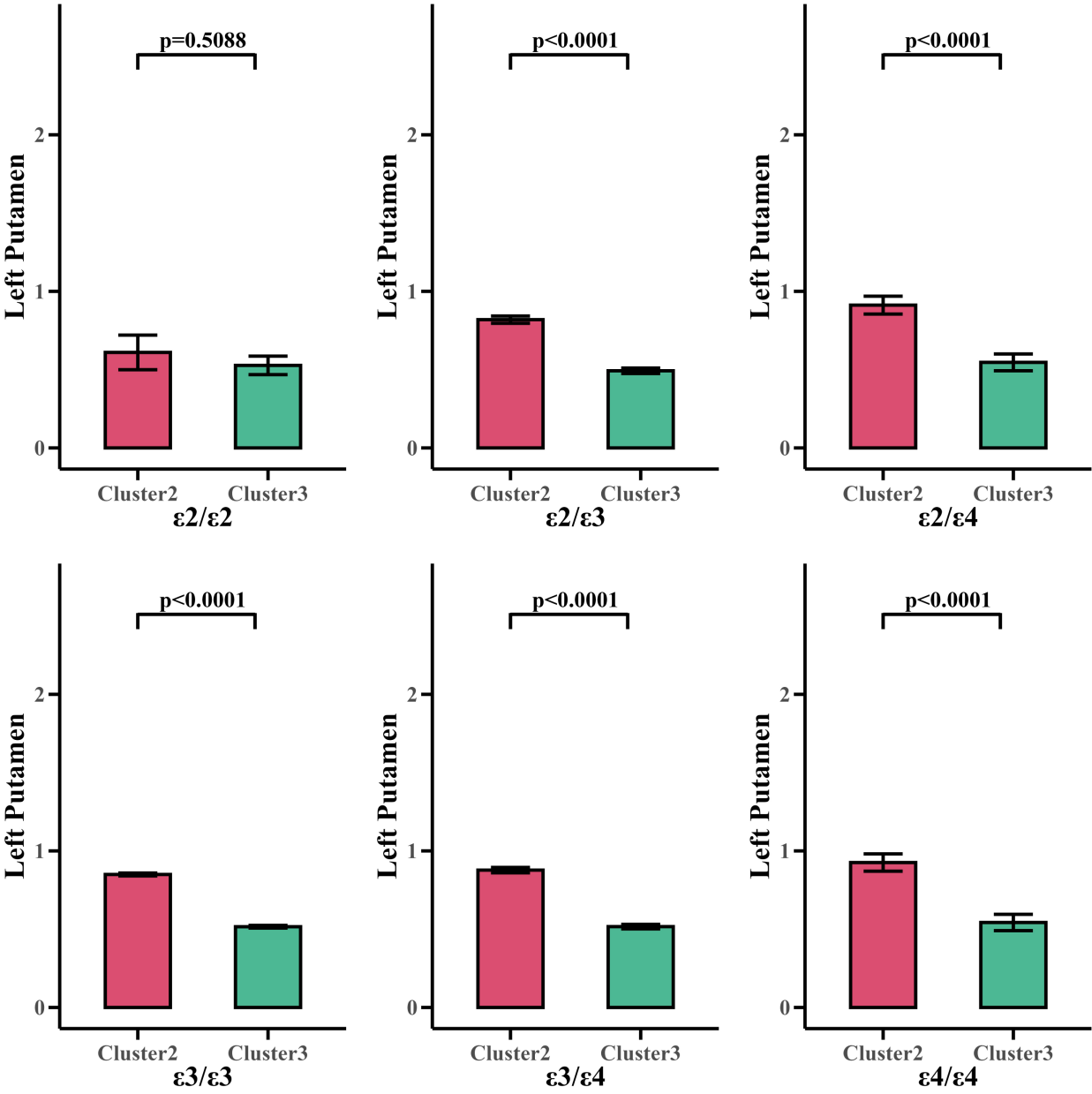

(iv)

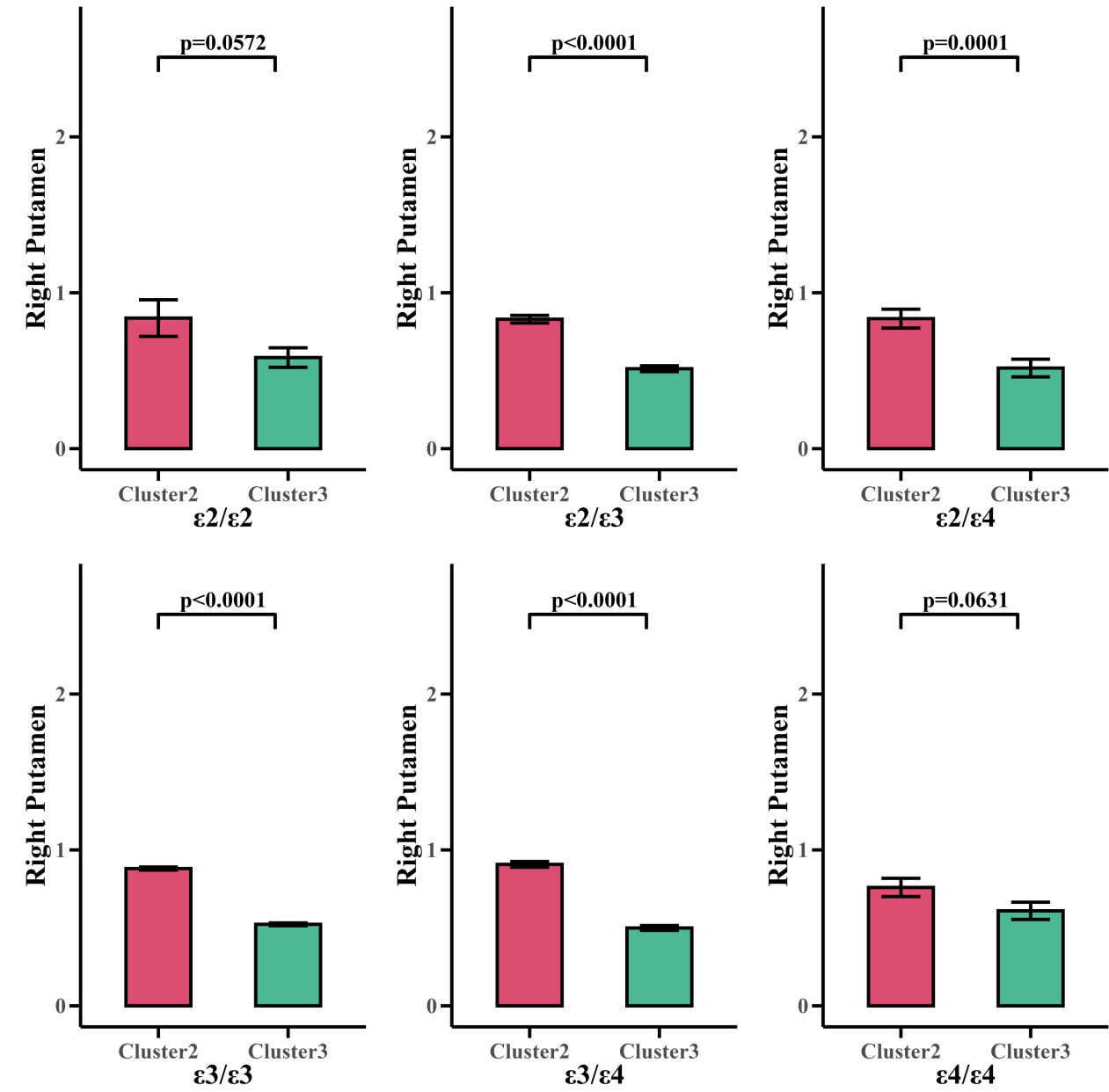

(v)

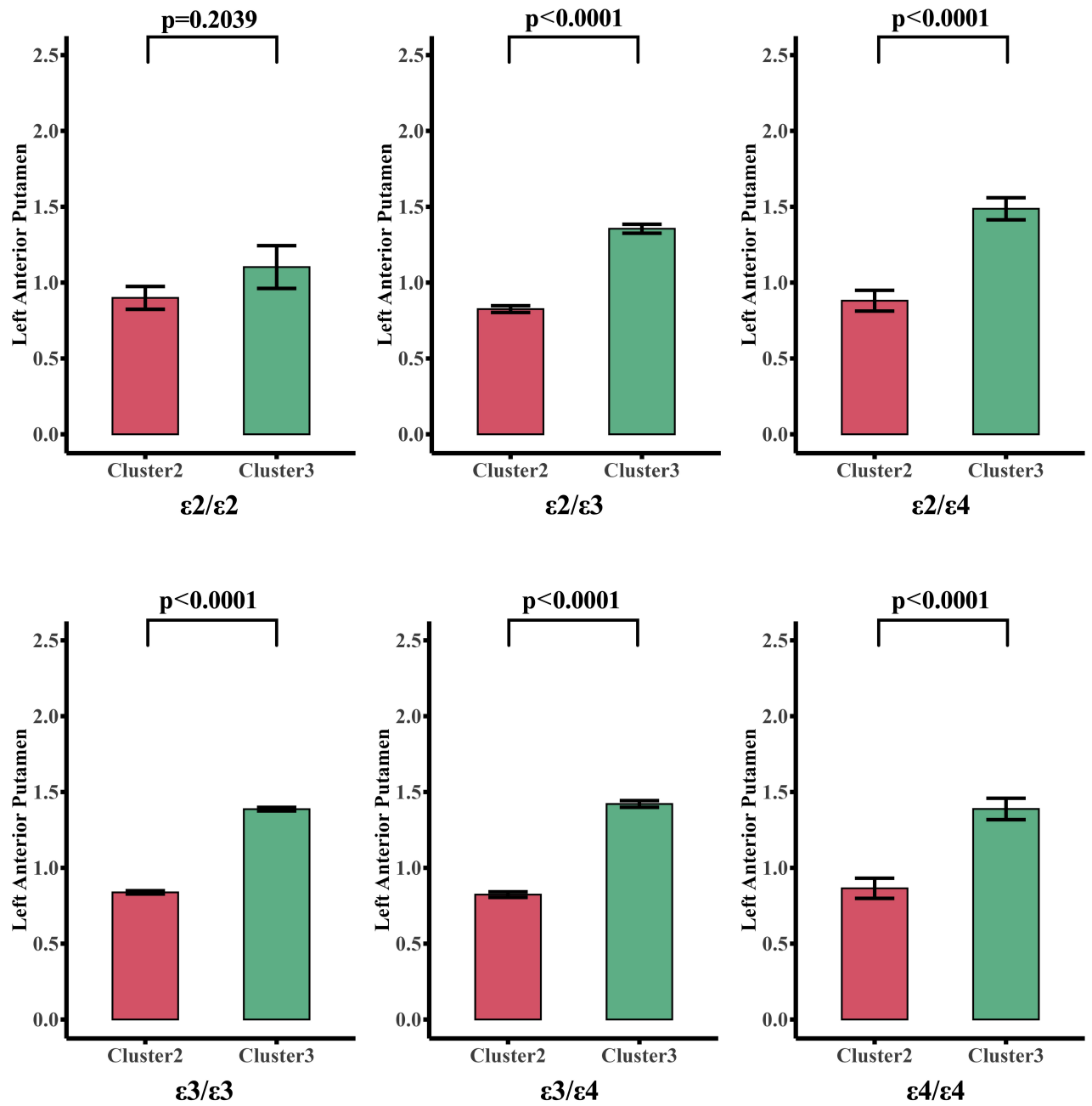

(vi)

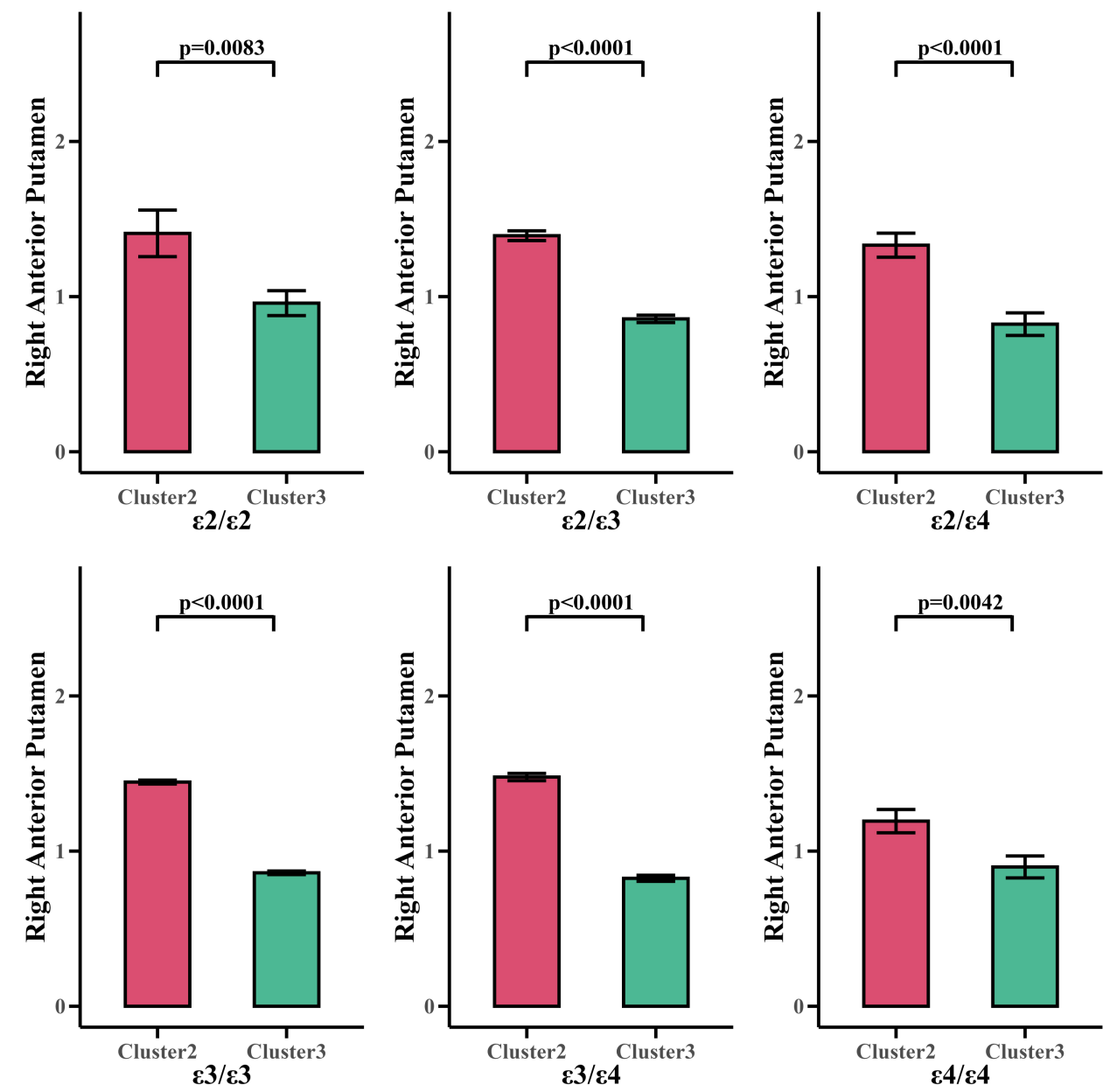

Supplement: Supplementary file 3 [file Image2.pdf]

## Supplementary Figure S3. Grey Matter Levels Across Clusters

(i)

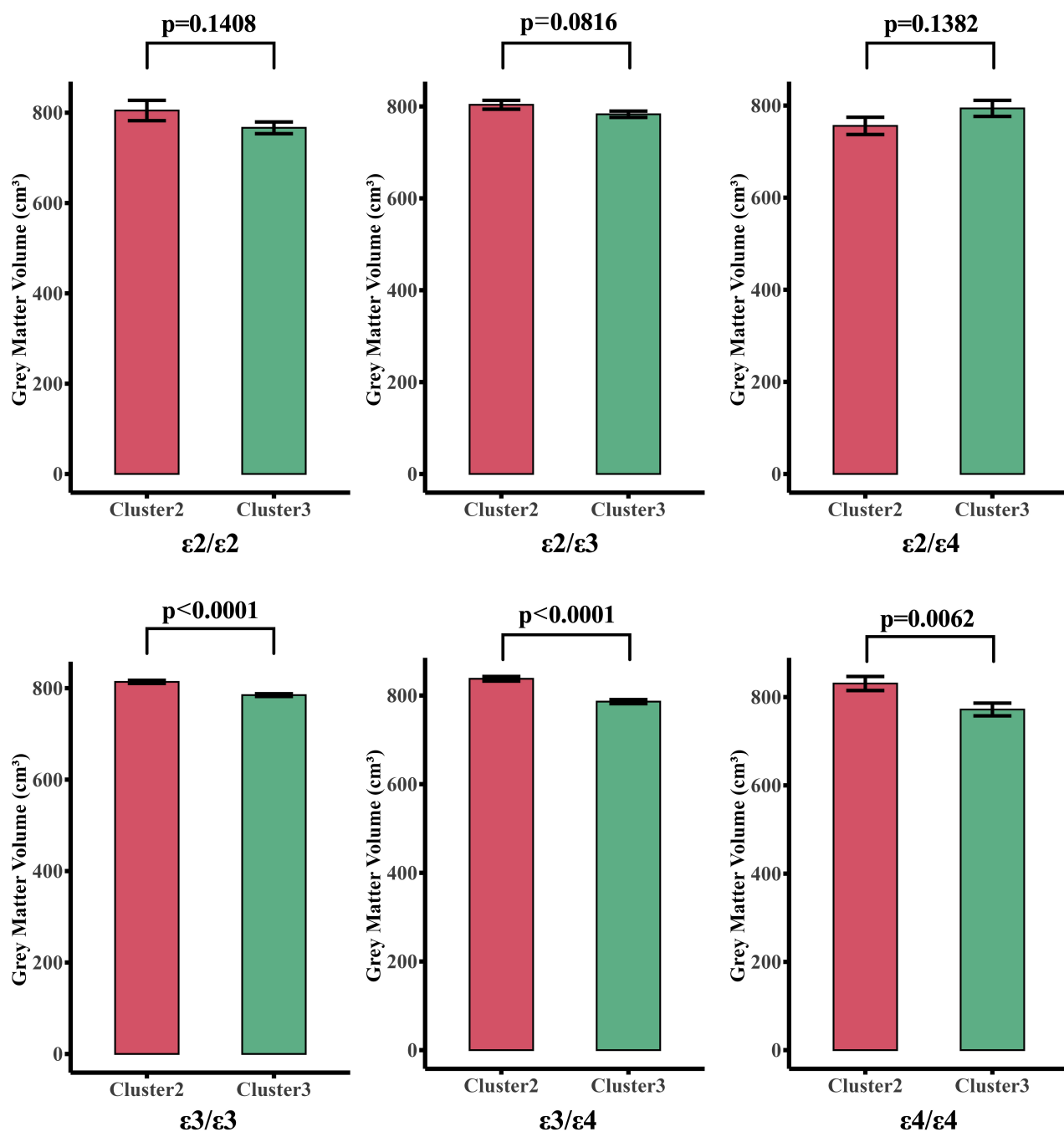

(ii)

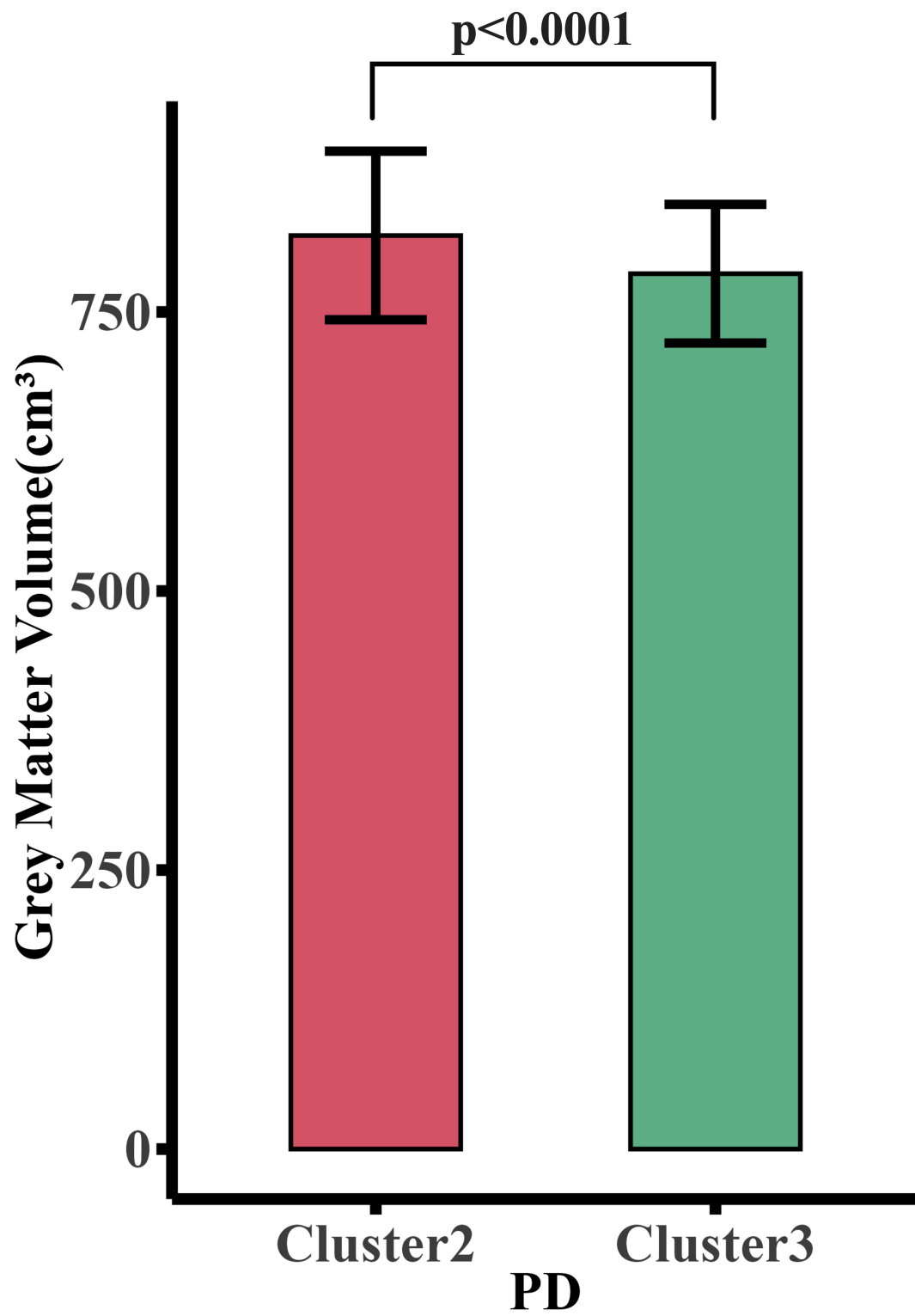

Supplement: Supplementary file 4 [file Image3.pdf]
